# Seasonality of Primary Small Bowel Volvulus and Its Variations Based on Sex and Place of Residence, North Western Ethiopia

**DOI:** 10.7759/cureus.27478

**Published:** 2022-07-30

**Authors:** Agegnehu B Bayeh, Belta A Abegaz

**Affiliations:** 1 Department of Surgery, Bahir Dar University, Bahir Dar, ETH; 2 Department of Biomedical Sciences, Bahir Dar University, Bahir Dar, ETH

**Keywords:** intestine, bowel obstruction, time series data, seasonality, primary small bowel volvulus

## Abstract

Background

Primary small bowel volvulus is a common surgical emergency in some parts of the globe. Its seasonal nature has not been widely researched. The main objective of this study was to assess its underlying patterns among different gender and geographical location.

Materials and methods

A hospital-based retrospective cross-sectional study was conducted from November 2020 to February 2021 at two comprehensive specialized hospitals in North West Ethiopia. The monthly count of primary small bowel volvulus was analyzed for patterns using Minitab 18. Graphical techniques such as run sequence plots, multiple box plots, and correlogram were used. Additive decomposition was also done. The degree of seasonal variation was measured in terms of seasonal indices generated for each month. A chi-square goodness-of-fit test at p < 0.05 was applied to determine statistical significance.

Results

A total of 235 patients were found to have surgically confirmed diagnosis of primary small bowel volvulus over six years. Most were males (77.4%) and from rural residence (73.2%). The mean age in years was 40.5 (±16.7). Overall, 179 (76.2%) of the total cases, 148 (81.3%) of males, and 138 (80.2%) of rural cases were admitted during June through November.

Conclusion

Seasonal variation was found to be a feature of primary small bowel volvulus. Knowing its seasonal nature helps raise the threshold of suspicion among health care providers to pass timely clinical decisions particularly in resource-limited setups.

## Introduction

Primary small bowel volvulus (PSBV) is a twist of the entire or part of the ileum and/or jejunum on its suspending mesentery [1,2] in otherwise normal peritoneal cavity [3-5]. It disproportionately affects part of the globe known as “the volvulus belt,” which includes parts of countries in Africa, Middle East, and Asia [6]. Its prevalence and incidence appear to vary widely. It is rarely encountered in Western countries [7-9]. A population-based study in the USA reported an incidence of small bowel volvulus of 1% [9]. Studies showed that it is a common surgical emergency in Ethiopia with a significant morbidity and mortality [10,11]. According to a study in North Central part of Ethiopia, more than half of small intestinal and a third of intestinal obstructions were caused by PSBV [10].

A delayed diagnosis and intervention lead to complications such as necrosis and perforation of the bowel. It may also lead to death [6,12,13].

It is hypothesized that it occurs as a result of contributions from different predisposing factors [14]. Seasonal variations in its occurrence were reported [15]. A study on Afghans showed that PSBV cases were higher during Ramadan [15]. A voluminous diet of high residue taken after a period of fasting was hypothesized to initiate the twist [14,15]. A study in northern Uganda showed that taking a greater volume of locally known drink called “Kongo” beer was related to the development of PSBV due to its rich serotonin content [16]. Longer small bowel mesenteric length and short mesenteric root were also hypothesized and reported as anatomic predisposing factors to allow abnormal mobility of the entire or a segment of the small bowel [2,14].

The main objective of this study was to assess PSBV for seasonal fluctuations based on sex and place of residence.

## Materials and methods

Study design, area, and period

A retrospective cross-sectional study was conducted from November 2020 to February 2021 at Felege Hiwot Comprehensive Specialized Hospital (FHCSH) and Tibebe Ghion Comprehensive Specialized Hospital (TGCSH) in North Western Ethiopia.

Ethics approval and consent to participate

Ethical approval was obtained from the Institutional Review Board of College of Medicine and Health Sciences, Bahir Dar University. An official permission was also obtained from the offices for the medical directors of FHCSH and TGCSH. Due to the retrospective nature of the study, no informed written consent was obtained from cases.

Study population

The study included clinical charts of all clients admitted and treated for intra-operatively confirmed primary small bowel volvulus at FHCSH and TGCSH from January 2015 to December 2020.

Data quality assurance and data collectors

Data collectors were four general practitioners who were given adequate training on the objectives of the study and the procedures to be adhered during data collection. The entire data collection process was supervised by the principal investigators, and a predeveloped data abstracting format was used.

Data collection procedures

The surgical records of all clients in the surgical departments and wards of the two hospitals were reviewed for a post-operative and discharge diagnosis of PSBV. Using the unique and specific medical registration number given to each client by the hospitals, the clinical charts of those with diagnosis of PSBV were retrieved from the medical registration management offices. Medical registration number, age, sex, place of residence, date of admission, date of operation, and intra-operative findings of 235 cases with surgically confirmed diagnosis of PSBV were recorded in a data abstracting format. Using the date of admission, the cases were grouped and recorded for each of the 72 months of the time span from January 2015 to December 2020.

Statistical analysis

The collected data were entered into Minitab 18 for analysis. The monthly count of PSBV was organized based on sex and different geographical location and analyzed for patterns of time series data using different graphical techniques such as time series plots, multiple box plots, and autocorrelation function plots. An additive decomposition of time series data analysis was also done. The degree of seasonal variation was measured in terms of seasonal indices generated for each of the months. A chi-square goodness-of-fit test at p < 0.05 was applied to determine statistical significance.

## Results

Sociodemographic data

A total of 235 cases were found to have surgically confirmed diagnosis of PSBV; of them, 182 (77.4%) and 172 (73.2%) were males and rural cases, respectively. The mean age in years was 40.5 (±16.7).

Seasonality of primary small bowel volvulus

A greater number of PSBV cases were admitted during June through November. Overall, 179 (76.2%) of the total cases, 148 (81.3%) of males, and 138 (80.2%) of rural cases were admitted during June through November (Table 1).

**Table 1:** Number of primary small bowel volvulus cases by month, sex, and place of residence from January 2015 to December 2020 at FHCSH and TGCSH, North West Ethiopia. FHCSH, Felege Hiwot Comprehensive Specialized Hospital; TGCSH, Tibebe Ghion Comprehensive Specialized Hospital

Cases	Month												Total
	Jan	Feb	Mar	Apr	May	Jun	July	Aug	Sep	Oct	Nov	Dec	
Total	9	11	9	7	8	21	30	32	36	36	24	12	235
Males	7	8	4	3	5	17	26	27	31	29	18	7	182
Females	2	3	5	4	3	4	4	5	5	7	6	5	53
Rural	7	6	5	4	4	15	21	27	30	28	17	8	172
Urban	2	5	4	3	4	6	9	5	6	8	7	4	63

**Figure 1:**
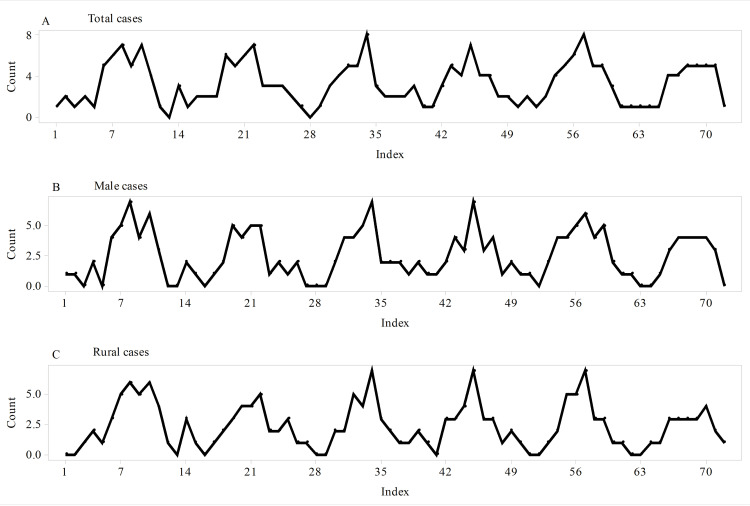
Time series plots of the total, male, and rural cases of primary small bowel volvulus. (A) Time series plot of total cases. (B) Time series plot of male cases. (C) Time series plot of rural cases. In all the three groups of primary small bowel volvulus cases, predictable repeating patterns with no tendency to increase over time were found to be features of the time series plots.

A time series plot for the total cases showed repeating patterns with no tendency to increase over time (Figure 1A). Similar patterns were also found for male and rural cases (Figures 1B, 1C).

**Figure 2:**
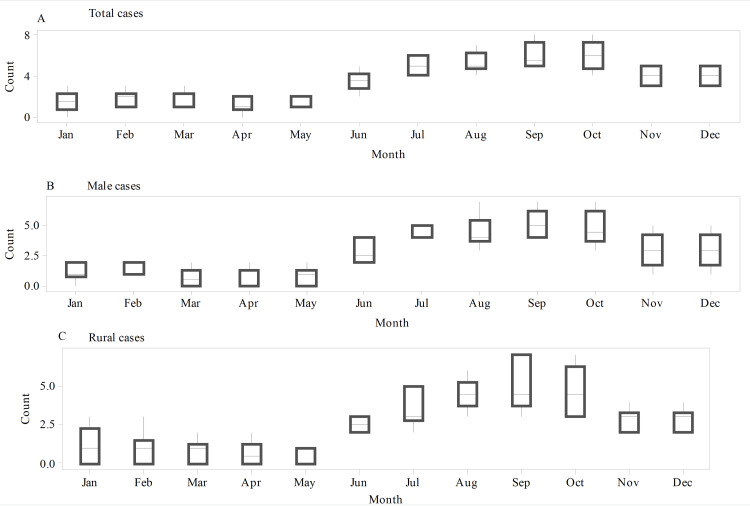
Multiple boxplots of primary small bowel volvulus admissions for the total, male, and rural cases. (A) Multiple boxplots of total cases. (B) Multiple boxplots of male cases. (C) Multiple boxplots of rural cases. The average number of admitted cases was generally higher during the rainy months (June through November) for total, male, and rural cases.

The average number of total cases increases from May through October. The highest and lowest average admissions were during October and April, respectively (Figure 2A). In males, the average admission peaks in September and gets to its nadir in March (Figure 2B). For rural cases, the highest average number of admitted cases was witnessed during August, September, and October, and the lowest was recorded in April (Figure 2C).

**Figure 3:**
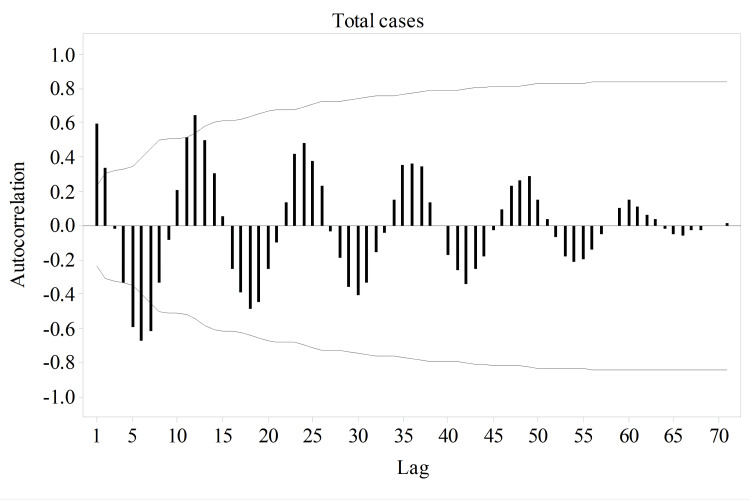
Autocorrelation plot of the total cases of primary small bowel volvulus. The presence of strong positive spikes at the seasonal lags shows that seasonal variation is a feature of primary small bowel volvulus.

The visible difference between the time series plots of the original and seasonally adjusted data and the presence of strong positive autocorrelations at the seasonal lags reflected that PSBV has a seasonal variation in its occurrence in the total cases (Figure 3).

**Figure 4:**
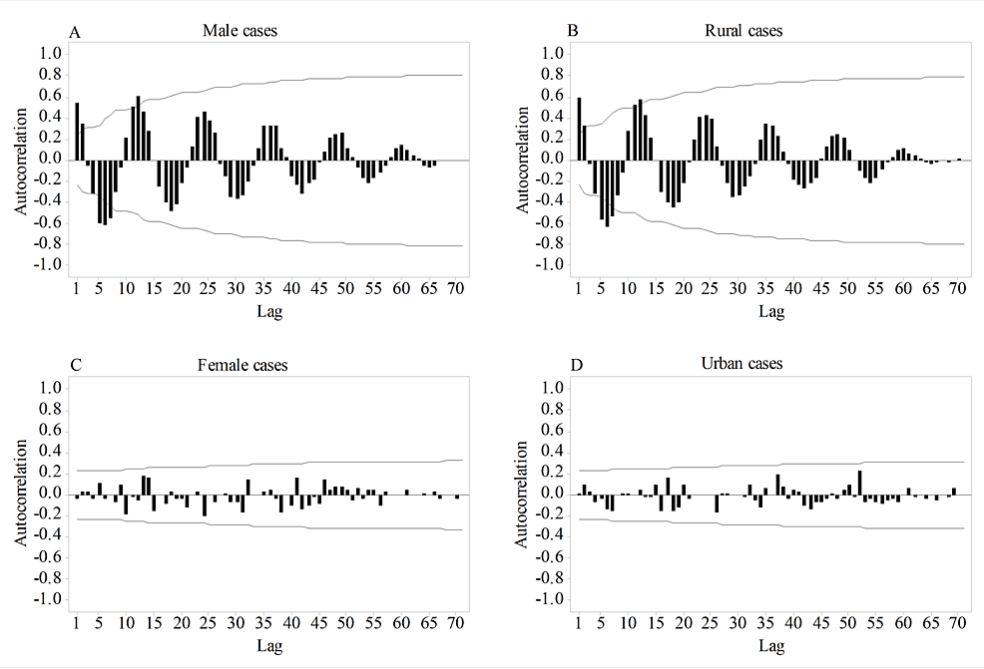
Autocorrelation plots of primary small bowel volvulus cases based on sex and place of residence. (A) Autocorrelation plot of male cases. (B) Autocorrelation plot of rural cases. (C) Autocorrelation plot of female cases. (D) Autocorrelation plot of urban cases. The presence of strong positive spikes at the seasonal lags shows the seasonal nature of primary small bowel volvulus in male and rural cases. On the other hand, the absence of such spikes in female and urban cases reveals that no seasonal variation exists.

Similar to the one for the total cases, autocorrelation function plots for males and rural cases showed the presence of strong positive autocorrelations at the seasonal lags, which reflects that seasonality is also a feature of PSBV in males and rural cases (Figures 4A, 4B); however, as evidenced by the absence of a significant autocorrelation at any of the lags, PSBV did not show seasonal pattern in female and urban cases (Figures 4C, 4D).

A chi-square goodness-of-fit test showed that the observed counts of primary small bowel volvulus for each of the months were significantly different from the expected counts for the total cases ($\chi$​^2^ (11) = 75.111, p < 0.001), males ($\chi$​​​​​​​​^2^ (11) = 83.846, p < 0.001), and rural cases ($\chi$​​​​​​​^2^ (11) = 77.349, p < 0.001). The observed values for the months of the winter (June, July, and August) and spring (September, October, and November) were significantly higher than the expected values. On the other hand, observed counts significantly lower than the expected counts were found for the months of the summer (March, April, and May) and autumn (December, January, and February).

**Figure 5:**
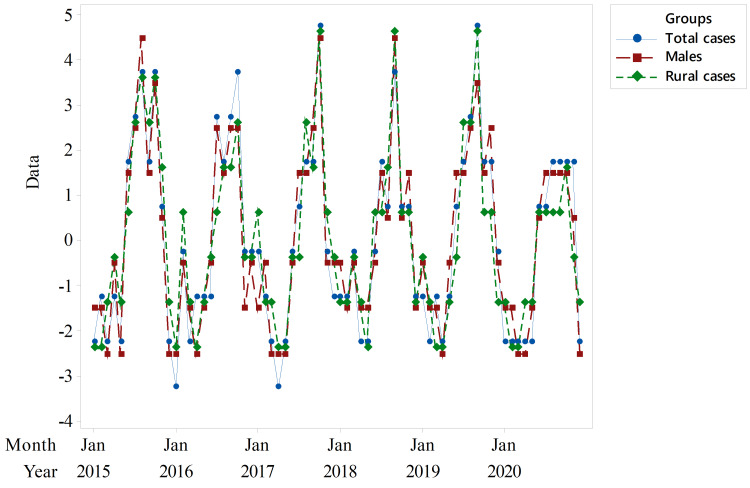
Seasonal components of primary small bowel volvulus among the total, male, and rural cases. It shows a close resemblance of the patterns among the three groups.

An additive decomposition showed that the underlying seasonal patterns of PSBV have a high level of resemblance among the total, male, and rural cases (Figure 5).

Seasonal indices revealed that the number of cases admitted were above the average during July through November among the total, male, and rural cases (Table 2).

**Table 2:** Seasonal indices based on groups for primary small bowel volvulus from January 2015 to December 2020 at FHCSH and TGCSH, North West Ethiopia FHCSH, Felege Hiwot Comprehensive Specialized Hospital; TGCSH, Tibebe Ghion Comprehensive Specialized Hospital

Patient group	Month											
	Jan	Feb	Mar	Apr	May	June	July	Aug	Sep	Oct	Nov	Dec
Total	-1.30	-1.30	-2.13	-2.26	-2.01	-0.13	1.83	2.00	2.49	3.49	0.45	-1.13
Males	-1.16	-1.57	-1.74	-2.32	-1.28	-0.20	1.59	1.64	2.68	2.72	0.34	-0.70
Rural	-1.31	-1.35	-1.35	-2.23	-1.40	-0.10	0.73	2.73	2.02	2.77	0.69	-1.19

## Discussion

A total of 235 cases were admitted for primary small bowel volvulus from January 2015 to December 2020. These patients were predominantly males and from rural areas. Most of them were young adult farmers. These findings are consistent with results of previously published papers from areas where primary small bowel volvulus is common [10,11]. Awedew et al. reported that in a study in Northcentral Ethiopia, the majority of such cases were males and rural residents with a mean age of 41.14 years [10]. Unlike those in endemic areas, the cases reported from areas where the volvulus is not commonly encountered are elderly. A population-based study in the USA showed that the majority of small bowel volvulus cases were white females with a mean age of 66.0 years [9].

Studies and reports hypothesized that primary small bowel volvulus occurs as a result of contributions from different predisposing factors [14,15]. Duke and Yar [15] reported that admission of cases of primary small bowel volvulus showed a correlation with the month of Ramadan among Afghans. Admissions were found to be 9-10 times greater during this period when a single large meal following several hours of fasting is taken.

Differences in small intestine morphometry seen among individuals [17-20], such as long small bowel mesentery with narrow attachment, might also contribute to its development [2,14].

Seasonal fluctuation in the occurrence of primary small bowel volvulus was the main focus of this study. The study area gets its stormy rain during the summer from June to September. Starting from May to November, the farmers are expected to engage in strenuous farming activities. Preparing farming land, ploughing, digging, uprooting weeds, and harvesting are some of the activities. Irrigation-based agriculture is less practiced in the area. These outdoor farming activities in the region are vastly shouldered by males living in rural areas. Most of these activities require erect and bowing body positions and are carried out in areas of several hours of on-foot travel away from home with daily trip.

The average number of patients of primary small bowel volvulus in this study was found to be higher in the rainy months of the southern hemisphere (June through November) than in the dry months of December through May. The seasonal fluctuation of primary small bowel volvulus was seen not only in the total number of cases but also in males and/or rural dwellers; however, this seasonal variation was not found in female cases and urban residents. The prolonged erect and bowing positions and the single voluminous high-fiber diet after several hours of farming activities and fasting that the farmers’ experience might contribute a greater slice to the development of primary small bowel volvulus.

The absence of seasonal variation of primary small bowel volvulus in females and urban cases, groups largely or totally left away from outdoor farming activities, supports this possible explanation for the occurrence of the volvulus. In line with these findings, previous studies hypothesized and reported that a bulky high-fiber meal after prolonged fasting may predispose individuals to primary small bowel volvulus [14,15].

Knowing this seasonal nature of primary small bowel volvulus help health workers have a high index of suspicion and pass timely decisions including early referrals particularly in resource-limited setups where costly investigations are scarce.

 To assess the underling patterns of primary small bowel volvulus by grouping the patients into total, male, female, rural, and urban groups is one of the new findings and contributions of this study; however, authors of the study remain fully cognizant of limitations of the study that can stem from its retrospective nature.

Future studies should focus on prospective multi-center studies of the variations in seasonal activities and experiences of farmers to look for possible areas of interventions for lifestyle modifications for prevention purpose and to establish the exact cause(s) of primary small bowel volvulus.

## Conclusions

Primary small bowel volvulus showed seasonal variations among male rural farmers with higher cases occurring during the months of winter and spring of the Southern Hemisphere. This is likely to relate to the activities and dietary experiences of farmers during the farming period. Knowing the seasonal nature of the disease would help raise the level of suspicion among health care providers for timely decisions and early referrals particularly in resource-limited setups where availability of diagnostic investigations is scarce.
